# Predictors of adherence to a new erythropoiesis-stimulating agent inpatient ordering policy: A cross-sectional study

**DOI:** 10.1371/journal.pone.0188390

**Published:** 2017-11-28

**Authors:** Numan Alabdan, Yazed AlRuthia, Mary E. D. Yates, Ibrahim Sales, Christopher K. Finch, Joanna Q. Hudson

**Affiliations:** 1 Department of Pharmacy Practice, King Abdulaziz Medical City, Riyadh, Saudi Arabia; 2 Department of Clinical Pharmacy, College of Pharmacy, King Saud University, Riyadh, Saudi Arabia; 3 Department of Clinical Pharmacy, College of Pharmacy, University of Tennessee Health Science Center, Memphis, Tennessee, United States of America; 4 Department of Pharmacy, Methodist Germantown Hospital, Germantown, Tennessee, United States of America; 5 Department of Pharmacy, Methodist University Hospital, Memphis, Tennessee, United States of America; 6 Department of Medicine, Division of Nephrology, Methodist University Hospital, Memphis, Tennessee, United States of America; Universita degli Studi di Perugia, ITALY

## Abstract

**Background:**

Erythropoiesis-stimulating agents (ESAs) are recommended for treating anemia in patients with chronic kidney disease and end-stage renal disease. However, misappropriate and over-use of these agents can be costly and unnecessary in some settings.

**Objective:**

The primary aim was to identify predictors of adherence to a newly approved ESA inpatient ordering policy. The secondary aims were to evaluate the impact of a 5-day delay in the initiation of ESA therapy on ESA usage, hemoglobin (Hb) levels, and costs.

**Methods:**

This retrospective observational record review included a sample of adult patients admitted to four tertiary care hospitals from November 1, 2013 to August 31, 2014. Multivariable logistic and linear regression analyses were used to calculate the odds of adherence to the new ESA inpatient ordering policy and the impact of this policy on discharge Hb level, respectively.

**Results:**

A total of 242 patients were included. The majority of the prescribers (77%) adhered to the new ESA ordering policy. Hemoglobin (OR = 1.306; 95% CI: 1.03–1.65) and ferritin (OR = 3.91; 95% CI: 1.23–12.51) levels at admission and length of hospital stay were positively correlated with the odds of patients receiving ESAs after day 5 (OR = 1.12; 95% CI:1.05–1.20). Furthermore, adherence to the new policy did not have a significant impact on discharge Hb level (β = 0.02349; *P* = 0.895).

**Conclusions:**

Prescribers were adherent to a 5-day delay in the initiation of ESA therapy policy which resulted in a reduction in ESA usage, did not impact the discharge Hb levels, and was proven to be cost effective.

## Introduction

With the implementation of Medicare’s bundled prospective payment system for end-stage renal disease (ESRD) patients, dialysis centers and healthcare systems alike in the United States are eager to explore and identify areas of potential cost savings [1]. The most glaring targets are the procedures and/or medications accruing the highest rate of expenditures while maximizing patient safety and minimizing undertreatment and mortality. Inarguably, one of the largest expenses in the management of patients with ESRD is the administration of erythropoiesis-stimulating agents (ESAs). Approximately 80% of dialysis patients in the U.S. require ESAs on a chronic basis to manage their anemia [2].

There are three ESAs currently marketed in the U.S.: darbepoetin alfa, epoetin alfa, and methoxy polyethylene glycol-epoetin beta. Both darbepoetin and epoetin are indicated for the treatment of anemia due to chronic kidney disease and myelosuppressive chemotherapy [3,4]. Epoetin has an additional indication for the treatment of anemia due to human immunodeficiency virus (HIV) therapy and in the reduction of transfusion in elective surgery [4]. Methoxy polyethylene glycol-epoetin beta is indicated for the treatment of anemia associated with chronic kidney disease (CKD) [5]. This agent is relatively new in the U.S. and not as widely used as epoetin alfa and darbepoetin alfa [5]. For all ESAs, it is recommended that they be used cautiously due to the increased risk of stroke, serious cardiovascular events, and death in CKD patients with Hb levels targeted to greater than 12 g/dL [6–8]. The most recent clinical practice guidelines for managing anemia in patients with CKD recommend against use of ESAs in CKD patients with Hb levels ≥ 10 g/dL [9].

ESAs differ in their frequency of administration due to their half-lives: epoetin alfa is dosed 1–3 times per week with a half-life of 6.8 hours, darbepoetin alfa every 2 weeks with a half-life of 25.3 hours, and methoxy polyethylene glycol-epoetin beta every 4 weeks with a half-life of 134 hours [3–5]. Clinical efficacy among these agents was compared in multiple trials aiming to maintain hemoglobin levels within 10–13.5 g/dL and within +1 g/dL of the baseline hemoglobin value. It was determined that 66%–76% of methoxy polyethylene glycol-epoetin beta recipients, 67%–72% of epoetin alfa or beta recipients, and 72% of darbepoetin alfa recipients maintained an average hemoglobin level within ±1 g/dL of baseline values [10–13]. The financial and safety concerns with ESA use have resulted in implementation of ESA-related cost management programs, primarily in the outpatient setting, with varying degrees of success. Programs have focused on converting ESAs from intravenous (IV) to subcutaneous (SubQ) administration [14], converting from a shorter acting to a longer acting ESA (e.g. epoetin alfa to darbepoetin alfa) [15], implementing nurse-driven anemia management protocols [16], pharmacist-managed ESA clinics, pharmacist-led management protocols [17,18], and other strategies [19–22]. In the inpatient setting ESA use is often not based on established protocols. Brophy et al., determined that the length of hospitalization for most patients with ESRD on dialysis was between 4 and 7 days; however, only approximately 13% of patients were administered ESAs during that time period [23].

In 2011, an ESA ordering process policy that allowed initiation of ESAs only for patients with at least a 3-day length of stay was implemented in our inpatient hospital, Methodist Le Bonheur Healthcare (MLH). The effect of the delay in the initiation of epoetin alfa (Procrit^®^), the current ESA on formulary, on the discharge Hb level was evaluated. No significant changes in Hb levels were found among hospitalized patients. Thus, the MLH Pharmacy and Therapeutics (P&T) committee approved a new ESA ordering policy whereby ESAs could only be administered to patients who have been hospitalized for at least 5 days. Under this new ordering policy, the pharmacist was required to do the following: (1) verify that the first ESA dose would be administered no sooner than day 6 of hospitalization, (2) verify that the patient had no contraindications to the ESA therapy, such as uncontrolled blood pressure (>185/110 mm Hg), Hb > 12 g/dL, or Hb ≥ 10 g/dL for patients with cancer, active bleeding, or curative cancer, (3) contact the prescriber if contraindications exist, and (4) contact the prescriber to obtain an order for an iron supplement if the patient was not receiving one.

The primary aim of this study was to identify predictors of adherence to the ESA inpatient ordering policy. The secondary aims were to evaluate the impact of the 5-day hold in ESA therapy on Hb levels and cost.

## Materials and methods

### Study design and data source

This was a retrospective observational record review study of patients receiving ESA therapy during hospitalization at MLH, which is a not-for-profit integrated seven-hospital healthcare delivery system with 1,689 licensed beds, based in Memphis, TN. The data was obtained via the review of electronic medical records at the MLH. The study was reviewed and approved by the University of Tennessee Health Science Center Institutional Review Board.

### Study population

A computer-based query of the electronic databases of the MLH pharmacy was obtained for adult patients 18 years or older who were prescribed ESAs between November 1, 2013, and August 31, 2014 (the time period after implementation of the 5-day hold). The computer query yielded 1210 records, of which 242 (20%) were selected randomly. This provided a sample of the populations that received ESAs during the 10-month evaluation period. Patients were divided into two groups based on whether ESAs were ordered following the ESA 5-day hold policy previously outlined (categorized as “adherent” or “non-adherent”). The following data were collected: (a) indication for ESA use; (b) sociodemographics (i.e., age, weight, and sex); (c) hospital length of stay (LOS); (d) history of bleeding at the time of ESA therapy initiation; (e) the first ESA dose and date of initiation; (f) whether iron therapy [administered orally (PO) or intravenously (IV)] was started before ESA initiation; (g) the number of packed red blood cell units received before ESA therapy initiation; (h) the number of hemodialysis sessions completed during hospitalization; (i) the completion of iron studies (i.e., iron, transferrin, ferritin); and (j) Hb levels on admission, at the time of ESA administration, and at discharge. This information was used to describe the groups and to evaluate predictors of adherence with the policy and outcomes as it relates to Hb and cost.

### Statistical analysis

Descriptive statistics for the patients’ characteristics were calculated using chi-square, two-sided Student’s t-test, and one-way analysis of variance (ANOVA), as indicated. Multivariable logistic regression analysis was conducted to examine whether adherence to the new ESA ordering policy was influenced by the ESRD diagnosis, hospital length of stay (LOS), admission Hb level, ferritin level at admission, and whether or not Hb level dropped by ≥2 g/dL within the first 5 days of admission. Furthermore, multivariable linear regression analysis was conducted to determine whether adherence to the new ESA ordering policy was associated with any significant change in Hb level at discharge, controlling for age, ESRD diagnosis, admission Hb level, and whether or not Hb level dropped by ≥2 g/dL within the first 5 days of admission. All statistical tests were performed at a significance level of 0.05 using SAS statistical software V9.4 (SAS Institute Inc., Cary, NC). The minimum sample size necessary for a medium effect size (f^2^ = 0.15) at power 0.80 and α 0.05 for multivariable linear regression that included 8 measured variables was 109 patients. For the multivariable logistic regression, the minimum sample size necessary for a medium effect size (odd ratio = 1.6) at power 0.80 and α 0.05 and two-tailed test was 232 patients. Therefore, 20% (242 patients) of the total hospitalized patient population during the time frame (November 2013 to August 2014) was selected to ensure adequate statistical power.

The number of ESA doses avoided and cost avoidance was determined using the average number of hemodialysis (HD) sessions from admission to initiation of an ESA in the sample population. This was done since ESAs are typically administered at MLH three times per week on HD days for HD patients. Cost data for ESA therapy was based on the per unit cost of epoetin alfa at the time of this evaluation ($0.010749/unit) and assuming an ESRD population requiring HD (since the majority of our patients requiring ESAs are ESRD patients) where:

Numberofdosesavoided=(averagenumberofHDsessionsxHDpatients)-numberofdispenseddoses

Costavoidance=numberofdosesavoidedxcostperdose

## Results

Data were collected for the 242 patients who were randomly selected. Table 1 shows patient characteristics for patients in the adherent and non-adherent groups. ESAs were ordered per policy in 187 of the 242 patients (77%). More than half of the patients had ESRD (69%) and were female (54%), with an average age of 61 years and weight of 86 kg. The mean Hb level on admission was slightly, but significantly lower among patients in the non-adherent group than in their counterparts (8.8 g/dL vs. 9.5 g/dL; *P* = 0.0036) (Table 1). Twenty percent of patients had a drop in their Hb level by ≥ 2 g/dL during the first 5 days of hospitalization with no significant difference between the adherent and non-adherent groups (21.4% vs. 18.2%; *P* = 0.605). The mean Hb level before the first dose of ESA was 8.4 g/dL with no significant difference between the adherent and non-adherent groups (8.4 g/dL vs. 8.3 g/dL; *P* = 0.841). The mean Hb level at discharge was 8.7 g/dL with no significant difference between the adherent and non-adherent groups (8.8 g/dL vs. 8.7 g/dL; *P* = 0.621). The average hospital LOS in the adherent group was significantly longer than in the non-adherent group (15.8 days vs. 9.3 days; *P*<0.0001). The difference between the mean number of days to initiation of ESA therapy was significant (3.7 days for patients in the non-adherent group vs. 8.8 days for the patients in the adherent group; *P*<0.0001). Furthermore, the difference between means of the number of hemodialysis sessions during hospitalization was also significant (0.66 sessions for patients in the non-adherent group vs. 2.36 sessions for the patients in the adherent group; *P*<0.0001). Eight percent of the patients received intravenous (IV) iron supplementation, and 20.3% received oral (PO) iron supplementation before the first ESA therapy. Thirty-four percent of the patients were given packed red blood cells (PRBCs) before they received ESA therapy, with patients in the adherent group receiving significantly more PRBC units than patients in the non-adherent group (0.86 units vs. 0.45 units, respectively; *P* = 0.0125). Forty-nine percent of patients had their ESA therapy started on the day of hemodialysis (Table 2).

**Table 1:** Patient characteristics at baseline.

Groups	ESA Ordering Policy Adherence			Total (n = 242)
	Non-adherent (n = 55)	Adherent (n = 187)	P-value	
**Male**	23 (41.8)	88 (47.1)	0.493	111 (45.9)
**Female**	32 (58.2)	99 (52.9)		131 (54.1)
**Age (yrs.)**	61.4 ± 14.7	61.0 ± 15.7	0.865	61.1 ± 15.5
**Weight (kg)**	83.8 ± 20.5	86.2 ± 29.2	0.490	85.7 ± 27.5
**Patients with CKD (not ESRD)**	17 (30.9)	59 (31.6)	0.931	76 (31.4)
**ESRD patients**	38 (69.1)	128 (68.5)	0.9282	166 (69.6)
**Bleeding on admission**	4 (7.3)	21 (11.2)	0.3967	25 (10.3)
**Cancer patients on chemotherapy**	1 (1.8)	5 (2.7)	0.7198	6 (2.5)
**Hb level on admission (g/dL)**	8.8 ± 1.4	9.5 ± 1.9	0.0036*	9.3 ± 1.8
**TSAT (<30%) on admission**	41(74.6)	14(25.5)	0.3865	55(22.7)
**Ferritin (<500 ng/ml) on admission**	51(92.7)	4(7.37)	0.1235	55(22.7)

Notes:

Data expressed as number (%) or mean ± standard deviation.

Hb: hemoglobin, ESA: erythropoiesis-stimulating agent, LOS: length of stay, CKD: Chronic Kidney Disease, ESRD: End Stage Renal Disease (requiring dialysis), IV: Intravenous, PO: orally, PRBCs: Packed Red Blood Cells.

* P-value <0.05; considered statistically significant.

**Table 2:** Patients characteristics during hospitalization.

Groups	ESA Ordering Policy Adherence			Total (n = 242)
	Non-adherent (n = 55)	Adherent (n = 187)	P-value	
**Hb dropped by ≥ 2 g/dL within the first 5 days**	10 (18.2)	40 (21.4)	0.605	50 (20.7)
**Hb level before the first dose of ESA (g/dL)**	8.3 ± 1.1	8.4 ± 1.1	0.841	8.4 ± 1.1
**Hb level at discharge (g/dL)**	8.7 ± 1.1	8.8 ± 1.14	0.621	8.7 ± 1.1
**Hospital LOS (days)**	9.3 ± 6.1	15.8±12.4	<0.0001*	14.3±11.6
**Day of hospitalization when ESA therapy was started**	3.7 ± 1.1	8.8 ± 5.2	<0.0001*	7.6 ± 5.1
**Number of HD sessions received during hospitalization for patients with ESRD**	0.79± 0.66	2.8 ± 2.1	<0.0001*	2.0 ± 2.2
**Number of HD sessions received during hospitalization for CKD patients (not ESRD)**	0.33±1.05	1.3 ± 2.4	<0.0001*	2.0 ± 2.12
**Patients receiving IV iron supplementation before ESA therapy**	5 (9.1)	16 (8.6)	0.9014	21 (8.7)
**Patients receiving PO iron supplementation before ESA therapy**	10 (18.2)	39 (20.9)	0.6645	49 (20.3)
**Patients receiving PRBCs before ESA therapy**	16 (29.1)	36 (36.4)	0.3193	84 (34.7)
**Number of PRBCs units received before ESA therapy**	0.453±0.822	0.856±1.510	0.0125*	0.76±1.39
**ESA started on the day of HD**	26 (47.3)	93 (49.7)	0.7484	119(49.3)

Notes:

Data expressed as number (%) or mean ± standard deviation.

Hb: hemoglobin, ESA: erythropoiesis-stimulating agent, LOS: length of stay, HD: Hemodialysis, CKD: Chronic Kidney Disease, ESRD: End Stage Renal Disease (requiring dialysis), IV: Intravenous, PO: orally, PRBCs: Packed Red Blood Cells.

* P-value <0.05; considered statistically significant.

For patients with higher Hb levels at admission (e.g., ≥9 g/dL), the odds of being in the ESA new ordering policy adherent group were 1.306 times larger than the odds for patients with lower Hb levels at admission (e.g., <9 g/dL) (OR = 1.306; 95% CI: 1.034–1.65; *P* = 0.0252). The odds for patients with long hospital LOS (e.g., >5 days) to be in the adherent group were 1.124 times larger than their counterparts with short hospital LOS (e.g., ≤5 days) (OR = 1.124; 95% CI: 1.053–1.20; *P* = 0.0005). Moreover, the odds for patients with higher ferritin levels (e.g., ≥ 500 ng/ml) were 4.18 times larger than their counterparts with lower ferritin levels (e.g., < 500 ng/ml) (OR = 3.91; 95% CI: 1.22–12.51; *P* = 0.0214) (Table 3). The new ESA ordering policy did not have a significant impact on the discharge Hb level (β = 0.023; 95% CI: -0.328–0.375; *P* = 0.8954). However, older age was associated with a higher Hb level at discharge (β = 0.016; 95% CI: 0.007–0.025; *P* = 0.0008). Moreover, higher hemoglobin level at admission was associated with higher hemoglobin level at discharge (β = 0.0988; 95% CI: 0.0016–0.196; *P* = 0.0464) (Table 4).

**Table 3:** Multivariable logistic regression of predictors for adherence to the new ESA ordering policy.

Variable	Odds Ratios (OR)	P-value	95% Confidence limits	
			Lower	Upper
**Hb level before the first dose of ESA**	0.82	0.3082	0.56	1.20
**Hb level at admission**	1.31	0.025*	1.03	1.65
**Hospital LOS**	1.24	0.0005*	1.05	1.20
**Ferritin level at admission**	3.91	0.021*	1.23	12.51
**Hb dropped by ≥ 2 g/dL within the first 5 days**	0.742	0.54	0.28	1.94
**ESRD patients**	1.58	0.23	0.75	3.33

Note:

* P-value <0.05; considered statistically significant.

**Table 4:** Multivariable linear regression for impact of the new ESA ordering policy on hemoglobin level at discharge.

Variable	Beta (β) estimate	P-value	95% Confidence limits	
			Lower	Upper
**ESA new ordering policy**	0.02349	0.8954	-0.328	0.375
**Hb level upon admission**	0.0988	0.0469	0.0016	0.196
**Age**	0.01603	0.0008*	0.007	0.025
**Hb dropped by ≥ 2 g/dL within the first 5 days**	-0.2666	0.2099	-0.6846	0.151
**Hemodialyzed patients with ESRD**	0.00422	0.979	-0.3157	0.324

Note:

* P-value <0.05; considered statistically significant.

Based on these data, 55 patients (23%) received an ESA before day 6, which equated to a total of 67 doses and 770,000 Units. The average dose was 11,493 Units and the average estimated cost per dose was $123.50. Therefore, the total cost of ESAs administered before day 6 was $8,277, which could have been saved if prescribers adhered with the new ordering policy for ESAs. The number of doses avoided, however, was 417. The estimated cost avoidance for these doses is $51,500 for the 10-month period, which equates to an annual estimated cost avoidance of $61,800.

## Discussion

Delaying the use of ESAs until the patient is discharged and resumes dialysis can prove lucrative to hospitals and healthcare centers in their efforts to minimize spending; however, the effect on clinical outcomes has been questioned [24]. The implementation of the new ESA ordering policy at our institution sought to curtail inappropriate prescribing of ESAs and increase cost savings, while not adversely affecting Hb levels. The results of this study suggest that such an ordering policy can achieve these goals without having a significant negative impact on the discharge Hb level. Adherence to this ESA ordering policy had many benefits and raised questions regarding the necessity of administering ESAs immediately on admission. Although the adherent group exceeded the 6-day delay period by nearly three days, there was not a significant difference between the groups when the mean Hb levels before the first dose of ESA and those at discharge were compared. We do acknowledge, however, that long-term follow up of Hb levels is necessary to fully assess the effect of withholding therapy due to the pharmacodynamics of ESAs (e.g. the delay in effect on Hb with a change in dose due to the red cell lifespan) [3–5].

The vast majority of practitioners adhered to the new ordering policy. The lack of adherence can be addressed through two direct methods: education and accounting for exceptions to the policy. Education of healthcare providers and adding an alert to remind pharmacists of the time requirement during order verification can enhance awareness of the new ordering policy. There are, however, situations where withholding therapy may not be deemed appropriate based on clinical judgment and this may be the reason for non-adherence to the new ESA ordering policy. For example, the mean Hb level upon admission was significantly lower in the non-adherent group, and 20% of patients experienced a ≥ 2 g/dL decline in their Hb levels within the first five days of hospitalization. These may be reasons practitioners would want to initiate therapy sooner than day six. Patients in the adherent group, however, had a significantly longer LOS than the non-adherent group, perhaps indicating more complex problems in this group.

One of the most common causes of hypo-responsiveness to ESAs is iron-deficiency anemia [25, 26]. Therefore, it is recommended to evaluate iron status in patients receiving ESAs. While more patients in the non-adherent group had suboptimal transferrin saturation and ferritin levels, there were no differences in the percent of patients who received oral or IV iron therapy before ESA initiation. With an average LOS of 14 days (SD ± 11.5) and time of ESA initiation of 7.4 days (SD ± 5), an evaluation of iron studies on admission may be warranted. Furthermore, iron therapy should be individualized based on objective values (e.g., TSAT < 30%, ferritin < 500 ng/mL) and the clinical status of the patient. Of note, a significantly higher number of units of PRBC were administered to patients prior to ESA therapy in the adherent group. Typically, PRBCs are administered in the setting of severe anemia when immediate correction of Hb is required or ESA use is ineffective.^24^ Although it is unknown whether this was done deliberately to delay the administration of ESAs, this finding highlights the need for concessions based upon clinical judgment.

Based on the findings from this ESA ordering policy, the recommendation to the MLH P&T committee was to continue the current policy of ESA initiation on day 6 of hospitalization. There are, however, some changes to consider. Pharmacists monitoring this policy should consider situations in which prescribers may want to deviate from this policy based on their clinical judgment. Such situations would include individualizing ESA therapy based upon the following scenarios: initiating ESA therapy in adult patients with stage 5 CKD requiring dialysis when Hb is > 9.0 and < 10.0 g/dL or CKD patients not requiring dialysis with Hb concentration <10.0 g/dL after considering the rate of decline in the Hb concentration, their prior response to iron therapy, the risk of needing a transfusion, the risks related to ESA therapy, and the presence of symptoms attributable to anemia.

Another recommended policy change to consider is a change in the Hb levels for which ESAs should be avoided in CKD from greater than 12 g/dL to greater than 11.5 g/dL to reflect KDIGO recommendations [24]. Lastly, the statement, “Epoetin should be started on day 6 of admission,” and the contraindications (CIs)/warnings of ESA therapy should be added on the order alert for pharmacist verification. It is also warranted to add the option to order iron studies and/or supplementation on the physician order alert. In addition, healthcare providers are in need of re-education regarding the ESA ordering policy and verification.

The limitations of this study should be considered when implementing hospital or health system specific ESA initiation policies. This was a retrospective study that evaluated a select group of patients receiving ESAs. Although the study sample might not accurately be representative of the larger population, we believe that the random selection of patients should reduce the sampling bias and make the sample more representative. However, including all the patient records in study sample should make the research findings more robust. There may also have been situations when initiation of ESAs prior to day 6 were reasonable; however, they would have been classified as non-adherent. In addition, other comorbid conditions, such as diabetes and hypertension, ESA indications other than ESRD, as well as the severity of illness were not controlled for in the analyses which could have contributed to the longer LOS among patients in the adherent group. Another limitation was the absence of structured educational sessions for healthcare providers regarding the policy. Finally, we were unable to follow patients after discharge (e.g., at dialysis centers) to assess the effect of this policy on Hb levels and ESA dose requirements following hospitalization. Therefore, the findings of this study have limited generalizability. However, despite these limitations, this evaluation did provide us with data to assess our use of ESAs and the clinical and cost implications. An annual cost savings over $60,000, due to the reduced use of ESA, is substantial for many institutions.

In conclusion, this ESA inpatient ordering policy was associated with an overall delay in ESA utilization by approximately three days, which resulted in a substantial cost avoidance. Future studies are needed to confirm the validity of these results from both a clinical and financial perspective.

## Supporting information

S1 De-identified Data (XLSX)
